# Positive bacterial culture in conjunctival sac before cataract surgery with night stay is related to diabetes mellitus

**DOI:** 10.1186/s12886-017-0413-7

**Published:** 2017-02-20

**Authors:** Tetsuhiro Kawata, Toshihiko Matsuo

**Affiliations:** 1 Department Ophthalmology, Okayama University Medical School and Graduate School of Medicine, Dentistry, and Pharmaceutical Sciences, 2-5-1 Shikata-cho, Okayama City, 700-8558 Japan; 2 Department of Ophthalmology, Fukuyama City Hospital, Fukuyama City, Japan

**Keywords:** Conjunctival sac culture, Cataract surgery, Diabetes mellitus, History of cancer, History of hospitalization

## Abstract

**Background:**

The aim of this study is to elucidate background clinical factors in patients with positive bacterial culture for the conjunctival sac before cataract surgery in Japan.

**Methods:**

Retrospective review was made on medical records of 576 consecutive patients who underwent conjunctival sac culture before cataract surgery with night stay at a hospital in 2 years from January 2013 to December 2014. In the patients with sequential bilateral surgeries, the data were chosen for bacterial culture in the eye which had earlier surgery. The age at surgery ranged from 33 to 100 years (mean, 76.7 years). Clinical factors, analyzed in relation with positive or negative bacterial culture, included the sex, the age, the presence of hypertension or diabetes mellitus, history of cancer, and history of hospital-based surgery at other specialties.

**Results:**

Bacterial culture of the conjunctival sac was positive in 168 patients while negative in 408 patients. In multiple regression analysis, the positive bacterial culture was related with the older age (*P* = 0.01), the presence of diabetes mellitus (*P* = 0.004), and the history of hospital-based surgery at other specialties (*P* = 0.001).

**Conclusions:**

Elderly patients with diabetes mellitus or previous hospital-based surgeries at other specialties have a higher rate of positive bacterial culture in the conjunctival sac before cataract surgery. This study would provide a hint for identifying patients at risk for carrying bacterial flora in the conjunctival sac.

## Background

Postoperative endophthalmitis is a rare but severe complication for intraocular surgeries such as cataract and glaucoma surgeries, and vitrectomy [1, 2]. To determine a patient at risk for postoperative infection, and hence, to reduce the rate of infection related with eye surgeries, bacterial culture from swab of conjunctival sac has been commonly done in preoperative assessment for intraocular surgeries [3–5] and refractive surgery [6]. In addition, conjunctival sac culture has been used to know changing patterns in the bacterial flora in part of the body.

So far to date, only a few studies have addressed which systemic clinical factors of patients were related with positive bacterial cultures of the conjunctival sac [7, 8]. In this study, we aimed to find systemic clinical factors which underlay culture-positive or culture-negative patients before cataract surgery in a city of Japan.

## Methods

### Patients

Retrospective review was made on medical records of 590 consecutive patients (248 men and 342 women) with 792 eyes who underwent conjunctival sac culture within 1 month (usually at 2 weeks) before cataract surgery at one night stay in Fukuyama City Hospital in 2 years from January 2013 to December 2014. In 202 patients with sequential bilateral surgeries, the data were chosen for bacterial culture in the eye which had earlier surgery. The second eye surgery was usually done at 1 week after the first eye surgery. The study was approved as a retrospective study by the institutional review board at Fukuyama City Hospital.

### Conjunctival sac culture

To obtain conjunctival sac culture, the lower conjunctival fornix was exposed by pulling the lower lid with a finger, and swabbed with a cotton (Rayon fiber) stick, and then, the cotton stick was dipped into agar medium for transport (BBL CultureSwab Plus Amies Medium Without Charcoal, BD, Becton, Dickinson and Company, Sparks, MD, USA). At clinical laboratories of Fukuyama City Hospital, the culture on sheep blood agar medium was continued for 48 h at 37 Celsius in an incubator with 5% carbon dioxide, and a single colony was considered as positive. On the occasion that two or more bacterial strains were cultured in 13 eyes of 13 patients, one strain in a largest amount was taken as responsible in consideration of background contamination.

### Inclusion and exclusion criteria

Fourteen patients were excluded from the analysis, based on the exclusion criteria: 1) eight patients with eight eyes which had preceding eye surgeries (one eye with strabismus surgery, one with pterygium resection, one with scleral buckling, one with vitrectomy, and four with intravitreous injection), 2) one patient who was involved in a clinical trial for chemotherapy, and 3) five patients with six eyes who had used antibiotic eye drops within 1 month prior to bacterial culture of the conjunctival sac due to ocular surface or eyelid infectious diseases, including keratitis, conjunctivitis, dacryocystitis, and blepharitis. After the exclusion, 576 patients remained in the analysis set.

Patients who underwent combined surgery, concurrent with cataract surgery, were not included in this study: 553 eyes of 531 patients with combined vitrectomy and 138 eyes of 123 patients with combined glaucoma surgery. Furthermore, in the 2-year period, cataract surgery was also done as day surgery in 1652 eyes of 1060 patients (551 men and 509 women with the mean age, 75.5 years, ranging from 21 to 101 years). These patients were not included in the analysis of this study because their electronic medical records had insufficient information on the history and medication. Night-stay surgery or day surgery was determined basically upon patients’ wishes. The age and the sex, as background factors, were not significantly different between the patients with night-stay surgery and day surgery.

### Clinical factors

Clinical factors which were collected and used for analysis in 576 patients, included the sex, the age, the presence of hypertension or diabetes mellitus, the history of cancer, the history of hospitalization for other diseases, and positive blood tests for syphilis, hepatitis B or C before cataract surgery. The presence of hypertension and diabetes mellitus was defined as taking hypotensive drugs and diabetic medications and/or insulin injection at the time of conjunctival culture, respectively. The history of cancer, of course, did not include the history of benign tumors. The history of hospitalization was narrowly designated as hospital-based surgeries at other specialties and taken positive only when patients had experienced hospitalization for surgeries at other specialties. Hospitalization for non-surgical treatment, such as intravenous drug administration or infusion, or hospitalization for examinations was not counted as the history of hospitalization in this study. Preoperative screening blood tests included serological test for syphilis (STS) and treponema pallidum latex agglutination (TPLA), hepatitis B surface antigen (HBsAg), and hepatitis C antibody (HCV-Ab).

### Cataract surgery

Cataract surgery was done from corneal incision or sclerocorneal incision on the superior side. The patients used 0.5% moxifloxacin eye drops four times daily for 3 days before the surgery, only after the results of conjunctival sac culture were obtained, irrespective of positive or negative bacterial culture. In the case that patients were using contact lenses, they were asked to stop wearing contact lenses 3 days before the surgery when prophylactic antibiotic eye drops were started. The use of eye drops for dry eye syndrome or glaucoma was not discontinued before the surgery.

At the beginning of the surgery, the ocular surface was washed with 16-time saline-diluted povidone iodine, and then, instilled with 0.3% ofloxacin gel. No intravenous antibiotics were given during the surgery. At the end of the surgery, the ocular surface was instilled with 1.5% levofloxacin eye drops, 0.3% ofloxacin and 0.1% betamethasone ointment. The patients were given cefcapene pivoxil 300 mg daily for 3 days after the surgery, and used 1.5% levofloxacin, 0.1% betamethasone, and 0.1% nepafenac eye drops four times daily for 1 month. Oral cefcapene pivoxil was not prescribed in patients with estimated glomerular filtration rate (eGFR) less than 45 mL/min/1.73 m^2^. No postoperative endophthalmitis was noted in the 2-year period of the study.

### Statistical analysis

The incidence of each clinical factor in two groups (culture-positive patients and culture-negative patients) were compared first by univariate analysis using chi-square test or Mann-Whitney *U*-test, and then compared by multivariate analysis using multiple regression analysis.

## Results

**Fig. 1:**
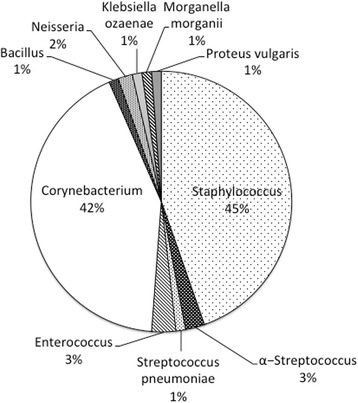
Pie chart, showing bacterial strains cultured from conjunctival sac in 168 patients before cataract surgery

The age of 576 patients (240 men and 336 women) at cataract surgery ranged from 33 to 100 years (mean, 76.7 years). Bacterial culture of the conjunctival sac was positive in 168 patients while negative in 408 patients. Table 1 shows the list of bacteria which were cultured from the conjunctival sac. Figure 1 shows a pie chart for bacterial strains. At cataract surgery, hypertension and diabetes mellitus were noted in 228 patients and 134 patients, respectively. Positive blood tests for screening of infectious diseases (syphilis, hepatitis B, hepatitis C) were found in 51 patients. In addition, 88 patients had history of cancer and 190 patients had history of hospital-based surgeries at other specialties.

**Table 1:** Bacteria cultured from conjunctival sac in 168 patients before cataract surgery

Classification	Bacteria	Number (%) of patients
Gram-positive cocci	Staphylococcus species	75 (44.6%)
	(Staphylococcus aureus)	(6)
	(MRSA)	(1)
	α-Streptococcus	4 (2.4%)
	Streptococcus pneumoniae	2 (1.2%)
	Enterococcus	5 (3.0%)
	(Enterococcus faecalis)	(1)
Gram-positive rods	Corynebacterium species	71 (42.3%)
	(Corynebacterium jeikeium)	(1)
	(Corynebacterium striatum)	(1)
	Bacillus	2 (1.2%)
Gram-negative cocci	Neisseria	3 (1.8%)
Gram-negative rods	Klebsiella ozaenae	2 (1.2%)
	Morganella morganii	2 (1.2%)
	Proteus vulgaris	2 (1.2%)
Total		168 (100%)

*MRSA* methicillin-resistant Staphylococcus aureus

In univariate analyses (Table 2), the age (mean, 78.8 years) of patients with positive bacterial culture of the conjunctival sac was significantly older than the age (mean, 75.9 years) of patients with negative culture (*P* = 0.002, Mann-Whitney *U*-test).

**Table 2:** Clinical factors related with positive or negative bacterial culture in conjunctival sac of 576 patients with cataract surgery

Clinical factors	Culture-positive patients (*n* = 168)	Culture-negative patients (*n* = 408)	Univariate *P* value	Multivariate *P* value
Age (years)				
Range (median)	39–99 (79.5)	33–100 (77)		
Mean	78.8	75.9	0.002	0.010
Sex				
Male	64 (38%)	176 (43%)		
Female	104 (62%)	232 (57%)	0.265	
Hypertension				
Yes	74 (44%)	154 (38%)		
No	94 (56%)	254 (62%)	0.160	
Diabetes mellitus				
Yes	50 (30%)	84 (21%)		
No	118 (70%)	324 (79%)	0.018	0.004
History of cancer				
Yes	29 (17%)	59 (14%)		
No	139 (83%)	349 (86%)	0.396	
History of hospital-based surgeries				
Yes	80 (48%)	110 (27%)		
No	88 (52%)	298 (73%)	0.001	0.001
Screening for infectious diseases				
Yes	18 (11%)	33 (8%)		
No	150 (89%)	375 (92%)	0.313	

Univariate analysis is done with chi-square test, except for the age (Mann-Whitney *U*-test). Multivariate *P* values are calculated by multiple regression analysis

The positive bacterial culture was also related with the presence of diabetes mellitus (*P* = 0.018, chi-square test), and the history of hospital-based surgeries at other specialties (*P* = 0.001, chi-square test). In multiple regression analysis (Table 2), the positive bacterial culture was related with the older age (*P* = 0.01), the presence of diabetes mellitus (*P* = 0.004), and the history of hospital-based surgeries at other specialties (*P* = 0.001).

## Discussion

This study revealed systemic clinical factors which underlay positive bacterial culture in the conjunctival sac of patients before cataract surgery with a night stay at a major hospital in a city with the population of 471 thousands, located in the western part of Japan. Older ages of patients, current suffering of diabetes mellitus, and the history of hospital-based surgeries at other specialties were three systemic factors which showed significant relation with the positive bacterial culture. These three factors in the patients have the common background which leads to immunologically compromised hosts. The diagnosis of diabetes mellitus in the patients of this study was strictly defined as currently having treatments as oral medication or insulin injection.

In preceding studies [7, 8], the old age of patients was found as a risk factor to have positive bacterial culture in the conjunctival sac before cataract surgeries. Furthermore, in one of the studies, patients with systemic risk factors, including diabetes mellitus, had a more chance to have positive bacterial culture of the conjunctival sac before intraocular surgeries [8]. Other series of preceding studies showed a high incidence of bacterial flora in patients with diabetes mellitus [9–11]. These preceding results, obtained in other countries, were consistent with the results in the present study which was conducted in a medium-sized city of Japan.

The bacteria which were detected in this study are considered to be part of the normal flora and rarely pathogenic. From the viewpoint of conjunctival bacterial flora, two major species, detected in the culture in this study, were Staphylococcus as a Gram-positive coccus and Corynebacterium as a Gram-positive rod (Table 1, Fig. 1). In the present study, patients with cataract surgery used moxifloxacin eye drops only for 3 days before the surgery. The prophylactic use of this antibiotic eye drop would be concluded as appropriate, based on the spectrum of moxifloxacin. The consecutive series of bacterial culture in the conjunctival sac in patients with cataract surgery would provide an opportunity to monitor the changing patterns of bacterial flora in the body. The monitoring would lead to an appropriate choice of antibiotics as eye drops at cataract surgery [12]. It should be noted that antibiotic-resistant bacteria, such as methicillin-resistant Staphylococcus aureus (MRSA), was detected only in one of the present series of patients. A recent regional care in a city of Japan to reduce antibiotics use might explain a low rate of MRSA detection in this study.

This study only included patients with cataract surgery at a night stay and excluded patients with day surgery. This inclusion criterion was derived from the fact that patients with hospital stay were, beforehand, screened for conjunctival flora, to avoid carrying and disseminating antibiotics-resistant bacteria in the hospital. This criterion would bring a potential selection bias in the study even though the night-stay surgery patients and day surgery patients did not show significant difference in background factors such as the age and the sex. Another potential selection bias in this study would be the exclusion of patients with combined surgeries, namely, cataract surgeries together with vitrectomy or glaucoma surgeries at one session. The patients with combined surgeries were excluded in this study simply because we aimed to focus on cataract surgeries which were a most frequent surgery in the hospital.

Another major limitation in this study is that the current use of medications was chosen as benchmarks for the presence of diabetes mellitus and hypertension in patients. These criteria would naturally miss diabetic and hypertensive patients who were not taking drugs at the time of cataract surgery. Along the same line, the use of antibiotic eye drops was used in this study to exclude patients with conjunctivitis or blepharitis which would influence the outcome of ocular surface culture. The use of medications was chosen as benchmarks to make the criteria simple and obvious in this retrospective study. Hemoglobin A1c, reliable marker for the diagnosis of diabetes mellitus, was not measured in all patients. Blood pressure at a single time point before the surgery was not reliable to make the diagnosis of hypertension. From the ophthalmic point of view, we did not pay attention to the use of eye drops for dry eye syndrome or the use of contact lenses which might also affect the ocular surface bacterial flora.

A narrow definition of previous hospital stay as hospital-based surgeries in this study is to focus on surgical intervention at other specialties. The duration of hospital stay for intravenous drug administration or examinations was usually short and sometimes difficult to differentiate from office-based or outpatient treatments or examinations. History of hospital-based surgeries at other specialties was obtained by interview with patients. The history was based on patients’ memory and included the episodes long time ago. Thus, the history of hospital-based surgeries in this study has a limitation from the standpoint of its accuracy and also of undefined time span between previous surgery and current cataract surgery.

## Conclusion

Elderly diabetic patients with previous history of hospital-based surgeries at other specialties would have a risk to have bacterial flora in the conjunctival sac. It is still controversial whether or not to use prophylactic antibiotic eye drops before cataract surgery. It is also controversial to screen bacterial flora in the conjunctival sac before cataract surgery. The present study would provide a hint for identifying patients at risk for carrying bacterial flora in the conjunctival sac.

## Abbreviations

HBsAg: Hepatitis B surface antigen
HCV-Ab: Hepatitis C antibody
MRSA: Methicillin-resistant Staphylococcus aureus
STS: Serological test for syphilis
TPLA: Treponema pallidum latex agglutination
